# Bioinformatics study of phytase from *Aspergillus niger* for use as feed additive in livestock feed

**DOI:** 10.1186/s43141-023-00600-y

**Published:** 2023-11-27

**Authors:** Hamdan Maulana, Yantyati Widyastuti, Nina Herlina, Abun Hasbuna, Aas Syiarudin Hasbi Al-Islahi, Lita Triratna, Novi Mayasari

**Affiliations:** 1 Faculty of Animal Husbandry, Department of Nutrition and Feed Technology, Universitas Padjadjaran, 45363 Jatinangor, Sumedang, West Java Indonesia; 2 National Research and Innovation Agency (BRIN), Research Center for Applied Microbiology, 16911 Cibinong, Bogor, West Java Indonesia; 3 PT. Berdikari Persero, 10110 Jakarta Pusat, Indonesia

**Keywords:** *A. niger*, Bioinformatics, Feed additive, Genetic engineering, Phytase

## Abstract

**Background:**

Phytase supplementation in rations can reduce their phytic acid composition in order to enhance their nutritional value. *Aspergillus niger* is a fungus that can encode phytase. This study aims to determine the characteristics of its DNA sequences and amino acid composition that encode the phytase enzyme, as well as to determine the primer designs.

**Method:**

This study used gene sequence data and protein-encoding phytase from *Aspergillus niger* that was collected manually from NCBI and PDB. The data was analyzed using SPDBV and then be aligned using the ClustalW Multiple Alignment features. The phylogenetic tree was built by Mega11 software. Primers were designed from selected candidate sequences that were analyzed. The designed primers were then simulated for PCR using FastPCR and SnapGene software.

**Results:**

There are 18 *Aspergillus niger* phytases in NCBI which is 14.87% of the total *Aspergillus*. There are 14 *Aspergillus niger* phytases that have identity above 95%. *Aspergillus niger* 110. M94550.1 is the closest strain to the PDB template. Candidate sources of phytase genes are *Aspergillus niger* 110.M94550.1, 48.2.BCMY01000003.1, and 92.JQ654450.1. The primer design has 2 possibilities of self-annealing and high melting temperature on the reverse primer. PCR simulation shows that the primer design can attach completely but still has the possibility of mispriming.

**Conclusion:**

This study suggests promising results for the future development of phytase enzyme production from *Aspergillus niger* as a feed additive using genetic engineering to enhance the quality of livestock feed in Indonesia.

## Background

Feed is the primary energy source for livestock to carry and support the maintenance and productivity [1]. Indonesia used feed from agricultural products and its by-products, which have high phytic acid concentration [2]. Phytic acid (myo-inositol hexakisphosphate) is a form of phosphorus storage in the feed crops such as grains, cereals and legumes [3]. Phytic acid is an antinutrient, especially for monogastric [4]. Phytic acid can bind other minerals such as Fe, Mg, Zn, Ca, and nutrition such as protein [4, 5]. Therefore, it might inhibit the absorption of the protein and minerals in the feed [3, 6]. Phytase supplementation is important for feed quality and therefore feed efficiency [7]. Phytase enzymes are still limited in Indonesia. In Indonesia, the technology, people, and material resources are still inadequate to produce their own phytase. However, Indonesia has the potential to produce phytase using native gene sources [8].

Phytase (myo-inositol hexakisphosphate phosphohydrolases) is an enzyme that catalyzes the release of phosphate from phytic acid [3, 8]. Phytase is widely used to overcome the problem of phytic acid and can be used as a feed additive for monogastric. Previous studies showed that phytase can improve nutrient absorption, especially P and Ca, reduce dependence on the use of mineral source feed additives, and reduce the environmental impact of monogastric due to the excretion of phytic acid in the feces [3, 7]. Phytase initiates stepwise removal of phosphate by decreasing their phosphorus excretion from phytate [3]. *A. niger*, *A. oryzae*, *F. venenatum*, *S. cereviciae*, *P. pastoris*, *K. lactis*, *P. griseoroseum*, and *E. coli* have been identified as host strain of phytases from fungal and bacteria isolates [9].

One of the methods to produce phytase is by genetic engineering using fungi. *Aspergillus niger* has been extensively reported in various literature as a fungus that can encode phytase (phyA and phyB) [8]. Based on its catalytic activity, *A. niger* phytase belongs to the histidine acid phosphatase (HAP) group [4, 10], which has activity under acidic conditions and is widely used as a hydrolyzer of phytic acid in animal feed [3]. The *Aspergillus* family has an active site motif -RHGXRXP- for catalysis and attachment and an -HD- motif for substrate attachment and product release [11]. The phytase production by fungi has been achieved through the use of solid-state and submerged fermentation methods [8, 12]. The first generation of commercial phytase was a fungal phytase from *Aspergillus niger* introduced in 1991 and marketed under the name Natuphos [13].

Bioinformatics study is an initial step of genetic and protein engineering [14]. This study allows some genetic engineering to be simulated by certain software and produce various kinds of data for genetic engineering [15, 16]. These data include the sequence and structure of genes and proteins, the level of kinship (phylogenetics), and the active site of the protein (enzyme). Therefore, this study aims to determine the characteristics of its DNA sequences and amino acid composition that encode the phytase enzyme, as well as the primer designs for it. Primer design can be used in further genetic engineering processes such as the isolation of target genes from the genome of the microbial gene source and identification of the presence of genes encoding target enzymes to show the potential of fungi in producing enzymes.

## Methods

### The 3D structure of phytase

The 3D structure of phytase was obtained from the Protein Data Bank (PDB) in (.pdb) format. The data obtained is information about the target enzyme, in this case, phytase from *A. niger*. The collected data was then combined with relevant literature for further study. The data was then analyzed using Swiss Protein Data Bank Viewer (SPDBV) software to determine the active sites and to observe the phytase enzyme protein structure in detail [14]. The obtained phytase protein sequences were then used as a template on sequence alignment and phylogenetic analysis.

### Sequence data collection

The data of *Aspergillus* was collected manually from the gene bank of the National Center of Biotechnology Information (NCBI) [14, 17]. Then the data is numbered based on the order in which the data was found. This helps us while analyzing DNA and protein sequences. The data used in this bioinformatics study is the coding region data (CDS) of DNA sequences and amino acids from *A. niger*. The sequence data was obtained and stored in the form of FASTA (.fas).

### Sequence alignment

Sequence alignment was performed using BioEdit software. *A. niger* DNA and protein sequence data will then be aligned using the ClustalW Multiple Alignment features [18]. The alignment data is then stored in the form of Genbank files (.gb) and FASTA (.fas).

### The phylogenetic tree

The phylogenetic tree was designed using the Mega11 software. The CDS sequence data that has been aligned and entered into the software will be processed using the construct/test maximum likelihood tree feature. The substitution method used in making this phylogenetic is the kimura parameter model 2 [19]. The kimura parameter model 2 was used for the substitution method in constructing this phylogenetic tree. The resulting tree will be analyzed to determine the degree of kinship between sequences. A high level of kinship and homology will be considered when selecting candidate sequences.

### Primer design

Primer design was made with the help of primer 3 + software using predetermined candidate sequences. Primer designs are made using complementary reverse start and end codons from the specified candidate CDS data. So, the *A. niger* phytase enzyme can be completely encoded. There are several criteria in making primers in this study, including the base pairs (bp) length between 18 and 30 bp and GC content between 45 and 60% [20]. The most optimal primers were then revalidated using oligocalculator software from http://biotools.nubic.northwestern.edu/OligoCalc.html. The software was set to identify hairpin and self-dimerization with a minimum of 3 bp.

### Primer simulation

The designed primers were then simulated for polymerase chain reaction (PCR) using FastPCR and SnapGene software [20, 21]. In silico simulation by FastPCR software is used to indicate the melting temperature, annealing temperature, and amplicon size that will be produced. FastPCR set by 0 mismatches to indicate the primers can anneal perfectly. SnapGene software is used to see the possibility of how primers anneal by showing the picture of PCR.

## Results

### 3D structure

**Fig. 1:**
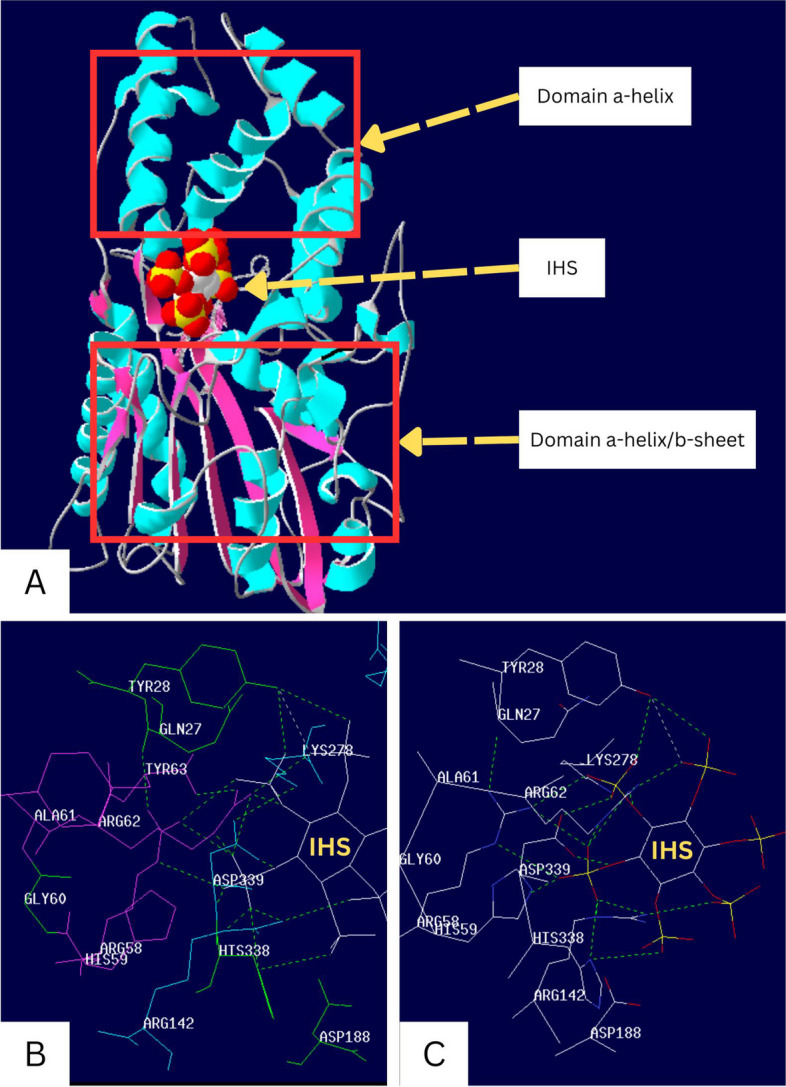
3D structure observation using SPDBV. **A** Secondary structure β-sheet shown by purple, α-helix by light blue, and loops by white (green on **C**). **B** The creatine phosphokinase (CPK) format consists of red for oxygen, blue for nitrogen, orange for phosphate, green for hydrogen bonds, and white to show amino acid molecular bonds

Based on PDB observations, we obtained the protein structure of phytase enzymes derived from *A. niger* 3k4q and 3k4p consisting of 444 amino acids. The protein secondary structure and peptide molecular structure accompanied by myo-Inositol hexasulfate (IHS) projection on the structure of *A. niger* 3k4q (Fig. 1). The active site is located in the gap between the large α-helix/β-sheet domain and the small α-helix domain (Fig. 1A). A close-up view of phytase (Fig. 1C) clarifies the location of the active site in the overall protein. Sulfate groups bind to Y28, R58, H59, R62, R142, K278, H338, and D339 residue (Fig. 1B). Therefore, there is no direct interaction between the enzyme and the inositol ring.

### Data collection

Based on *Aspergillus* phytase data contained in NCBI (data not shown). *Aspergillus* phytase consists of 121 data. There is 18 (14.87% total) of phytase data from 17 *A. niger* (Table 1). Some *A. niger* data were whole genome shotgun sequences and CDS with a range of 1404–2071 bp and 467–522aa originating from China and India while most of the data were not mentioned.

**Table 1:** Sequence characteristics based on *A. niger* 3k4p

No	Organism	Accession number, length		Product	Active site
		**DNA**	**Amino acid**		
48	*A. niger*, An76	BCMY01000003.1 2.393.285 bp	GAQ37582.1 522aa	Phytase B precursor	H78/D385
			GAQ37712.1 467aa	Phytase	H82/D362
49	*A. niger* An76	BCMY01000021.1 600.870 bp	GAQ46510.1 517aa	Phytase	H64/D363
85	*A. niger*	AB022700.1 1.515 bp	BAA74433.1 467aa	Phytase	H82/D362
92	*A. niger* strain MI 2	JQ654450.1 1.515 bp	AFJ79736 .1 467aa	Phytase	H82/D362
93	*A. niger* MI 1	JQ654449 .1 1.515 bp	AFJ79735.1 467aa	Phytase	H82/D362
94	*A. niger* strain N14	AY426977.1 1.525 bp	AAR08366.1 467aa	Phytase	H82/D362
95	*A. niger*	AY745739.1 1.506 bp	AAU93518.1 467aa	Phytase	H82/D362
97	*A. niger* var *awamori*	L02421.1 2.379 bp	AAA16898.1 467aa	Phytase	H82/D362
98	*A. niger* phytase	JQ241266.1 1.404 bp	AFE56108.1 467aa	Phytase	H82/D362
104	*A. niger*	EF197825.1 1.934 bp	ABM92786.1 467aa	Phytase A	H82/D362
107	*A. niger*	L20567.1 1.861 bp	AAA02934.1 479aa	Phytase b	H82/D338
108	*A. niger* phytase	AY513749.1 1.506 bp	AAS00648.1 467aa	Phytase A	H82/D362
110	*A. niger*	M94550.1 2.665 bp	AAA32705.1 467aa	Phytase	H82/D362
111	*A. niger*, NII 08121	JN196454.1 1.506 bp	AET71192.1 467aa	Phytase A	H82/D362
112	*A. niger*	AF218813.1 1.528 bp	AAF25481.1 467aa	Phytase precursor	H82/D362
113	*A. niger* var *awamori*	L02420.1 2.071 bp	AAA16897.1 479aa	Acid phosphatase	H82/D338
114	*A. niger* BCC18081	EU786167.1 1.404 bp	ACE79229.1 467aa	Phy A	H82/D362

*DNA* deoxyribonucleic acid

### Sequence alignment

**Fig. 2:**
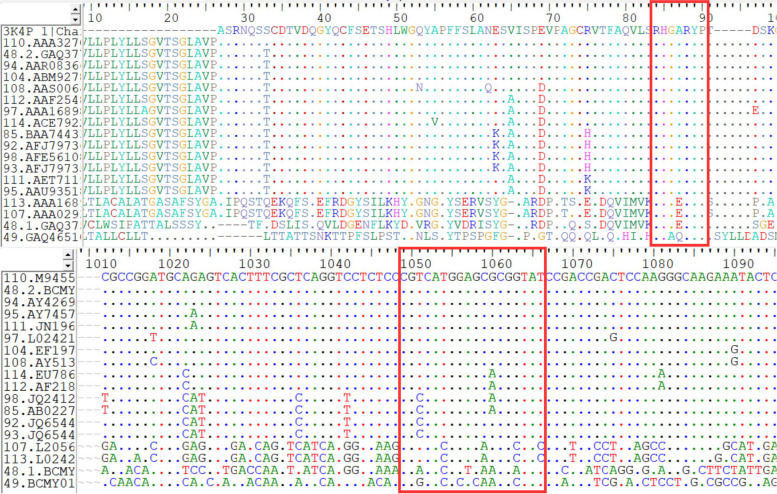
Diagram of the amino acid (top) and gene (bottom) alignment. The red box shows the active site motif in the sequence

In addition, multiple sequence alignments were performed to find a conserved motif in the DNA sequences. A total of 18 phytases were aligned with *A. niger* 3k4p as a base template (Fig. 2). *A. niger* phytase is well conserved as indicated by the dot mark. Characteristics sequence based on *A. niger* 3k4p also shows in (Table 1). There are 14 organisms with an identity to each other of 95% (Fig. 2). Phytase *A. niger* 3k4p has the closest kinship with phytase 110. AAA32705.1 and shows 95% identical. Phytase 113.AAA16897.1 and 107.AAA02934.1 had an identity with each other of 98.8% and were the most distantly related *A. niger* phytase followed by 48.1.GAQ37582.1 and 49.GAQ46510.1. Both have identities with templates below 22%. Based on the alignment of 14 *A. niger* phytase, the mutations were found in the phytase sequence (Table 2*)*.

**Table 2:** Mutated sequence fragment from *A. niger* phytase

Code	V276	S278	E284	T291	T293	S312	K317	D328	N332	K340	D409
110	*	*	*	*	*	*	*	*	*	*	*
48.2	*	*	*	S	*	N	*	E	*	*	*
94	*	*	*	S	*	N	*	E	*	*	*
104	*	*	*	S	S	N	N	E	*	*	*
108	*	*	*	S	S	N	N	E	*	*	E
112	*	T	*	S	*	*	*	*	*	*	*
97	A	*	*	*	*	*	*	*	H	*	*
114	*	T	*	S	*	*	*	*	*	*	*
85	*	*	*	S	*	*	*	E	*	N	*
92	*	*	*	*	*	*	*	E	*	N	*
98	*	*	*	S	*	*	*	E	*	N	*
93	*	*	*	*	*	*	*	E	*	N	*
111	*	*	*	*	*	*	*	*	*	*	*
95	*	*	G	S	*	*	*	*	*	*	*

^*^Conserved amino acids

### Phylogenetic tree analysis

**Fig. 3:**
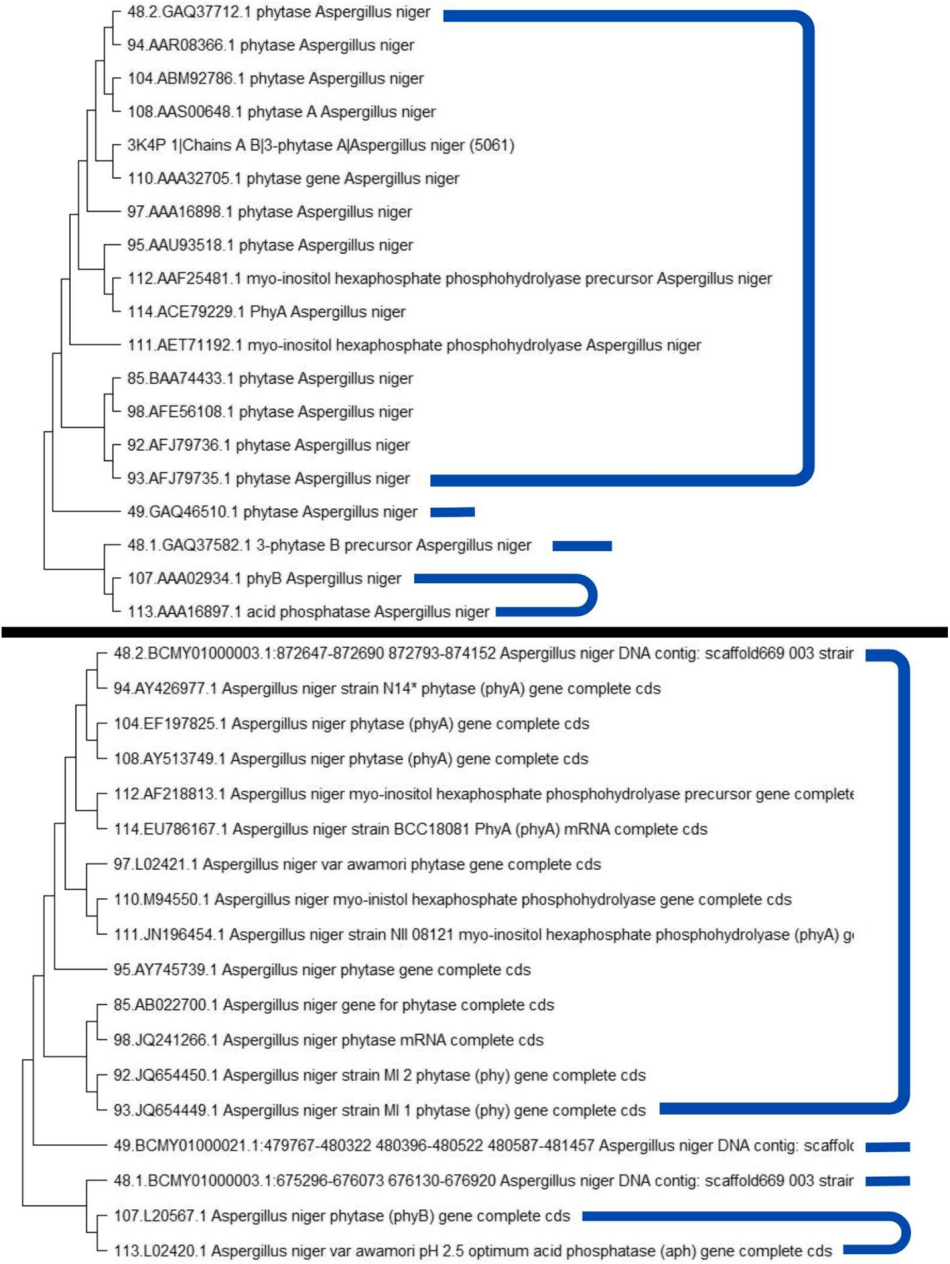
Phylogenetic tree based on amino acids (top) and genes (bottom)

The phylogenetic analysis showed the classification that aims to estimate the evolutionary relationship of an organism [22]. We found that both the DNA and amino acid sequence of *A. niger* phytase are divided into 4 groups as shown in (Fig. 3). *A. niger* 110.M94550.1 was the closest source to the template.

### Primer design

We selected three candidates based on kindship and homology of sequences. There are *A. niger* 110.M94550.1, 48.2.BCMY01000003.1, and 92.JQ654450.1 (Table 3). The complementary reverse start and end codons of the three primer designs are quite identical to each other. All secondary structures showed good results with only 2 and 3 possibilities of self-annealing and no hairpin possibility occurred (Table 4).

**Table 3:** Primer design results with Primer3 + (P3) and oligocalculator (Olc)

Code	Sequence	Base	Tm (°C)			GC content (%)	
			**P3 + **	**Olc**		**P3 + **	**Olc**
(….. primer forward…..)							
48.2	5′ATGGGCGTCTCTGCTGTTC3′	19 bp	61.4		59.5	57.9	58
92	5′ATGGGTGTCTCTGCCGTTC3′	19 bp	61.1		59.5	57.9	58
110	5′ATGGGCGTCTCTGCTGTTCTACTT3′	24 bp	64.7		65.2	50	50
(……primer reverse…..)							
48.2	5′CTAAGCAAAACACTCCGCCCAATC3′	24 bp	66.9		65.2	50	50
92	5′CTAAGCAAAACACTCCGCCCAATC3′	24 bp	66.9		65.2	50	50
110	5′CTAAGCAAAACACTCCGCCCAATC3′	24 bp	66.9		65.2	50	50

Oligocalculator (Olc), Primer 3 + (P3 +), melting temperature (Tm), Base pair (bp)

**Table 4:** Results of the analysis of primary secondary structures

Code	Primer 3 +		Oligocalculator			
	**SA**	**SC**	**Hairpin**	**SA**	**C**	**Visualization**
(……Primer forward…..)						
48.2	2	-	-	2	-	5′ ATGGGCGTCTCTGCTGTTC 3′ 3′ CTTGTCGTCTCTGCGGGTA 5′ 5′ ATGGGCGTCTCTGCTGTTC 3′ 3′ CTTGTCGTCTCTGCGGGTA 5′
92	2	-	-	-	-	-
110	2	-	-	2	-	5′ ATGGGCGTCTCTGCTGTTCTACTT 3′ 3′ TTCATCTTGTCGTCTCTGCGGGTA 5′ 5′ ATGGGCGTCTCTGCTGTTCTACTT 3′ 3′ TTCATCTTGTCGTCTCTGCGGGTA 5′
(……Primer reverse…..)						
48.2	3	-	-	-	-	-
92	3	-	-	-	-	-
110	3	-	-	-	-	-

*SA* self-annealing, *SC* self complementary, *C* complementary

### Primer simulation

**Fig. 4:**
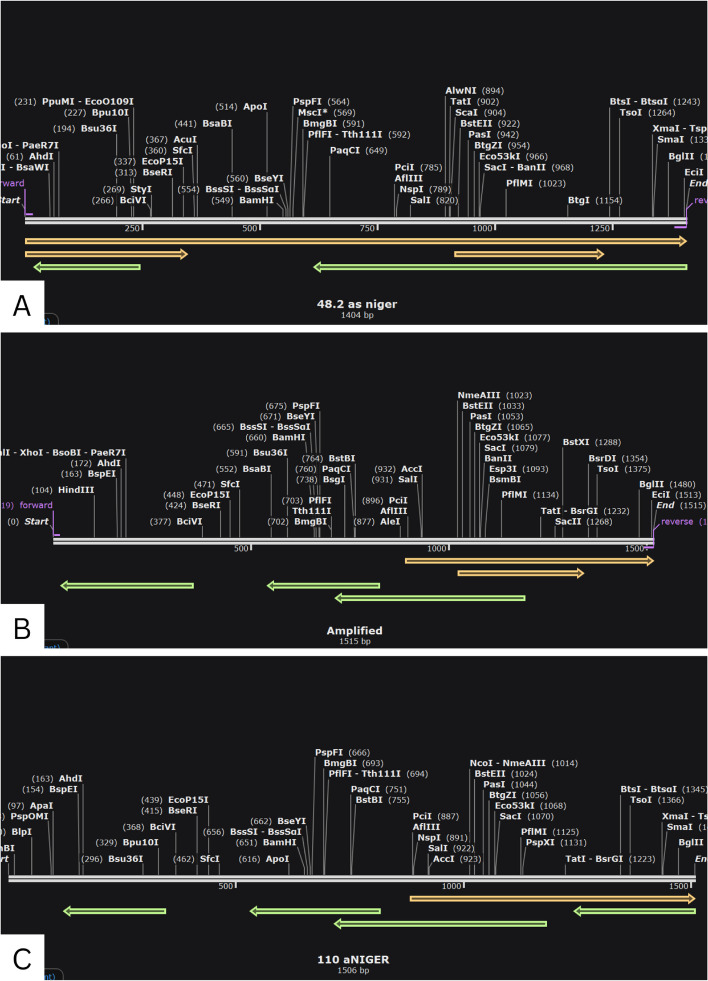
PCR simulation of Snapgene phytase *A. niger* 4.8.2.BCMY01000003.1 (**A**), 92.JQ654450.1 (**B**), and 110.M94550.1 (**C**). Forward primer (orange), reverse primer (green)

The PCR simulation using FastPCR shows that all three primers have a maximum amplicon size and high annealing temperature (Table 5). PCR simulation using Snapgene shows all primer designs still have the possibility of mispriming. This is indicated by the disconnected arrow (Fig. 4).

**Table 5:** Results of PCR simulation using FastPCR

Code	Primer	Position	% anneal	Tm (°C)	Ta (°C)	Amplikon size (product)
48.2	Forward	1–19	100	62.4	70	1404
	Reverse	1381–1404	100	64		
92	Forward	1–19	100	62.5	70	1515
	Reverse	1492–1515	100	64		
110	Forward	1–24	100	66.2	72	1506
	Reverse	1483–1506	100	64.0		

*PCR* polymerase chain reaction, *Tm* melting temperature, *Ta* annealing temperature

## Discussion

Enzymes are proteins that act as biocatalysts for a reaction. İt is a macromolecule composed of amino acids and is synthesized by nucleotides by encoding nitrogenous bases into amino acids. Phytase 3k4p has a complex structure and good stability shown by the dominance of the α-helix amino acid structure. The dominance of the α-helix structure indicates that the protein is non-polar/hydrophobic and results in good stability. A previous study reported that the α-helix domain plays a role in stabilizing amino acid residues in *A. niger* phytase [11]. The α-helix structure is a secondary structure that can improve thermal stability with a critical role in energy dissipation [23, 24]. However, the active site is stiffer than bacterial HAPs, because the α-helix on the C-terminus of the RHGXRXP-containing loop is shorter than bacterial HAPs [25]. Active site flexibility is considered a requirement for lowering the free energy barrier, improving active site accessibility, and accelerating catalytic efficiency [26].

The active site of phytase *A. niger* contains five disulfide bonds. Most of them are located on loops near the surface of the phytase protein [10]. Residues contact IHS via hydrogen bonds with all sulfate groups except 6-sulfate. IHS is an inhibitor used to analogize phytic acid or IHP. The IHS is isosteric and isoelectronic with myo-inositol hexaphosphate (IHP) [11]. The phytic acid in feed is attracted by hydrogen bonds and then interacts and reacts directly with amino acid residues through phosphate groups, thus forming a covalent phospho-histidine intermediate bond [25]. This process is then followed by hydrolysis and release of histidine residues.

Our present study found that *Aspergillus* can encode two phytases. This can be attributed to the fact that *A. niger* is a eukaryotic fungus. *A. niger* 48.1 is still in the form of phytase precursor and 48.2 is already in the form of phytase. This is possible because the first phytase carries a signal peptide that will determine the location of the expression of phytase, which is outside the cell [27]. So that the new phytase will be active after this signal peptide is released when it finishes delivering phytase to its place of expression. The second phytase will be expressed inside the cell.

Analysis of the amino acid sequences of gene and amino acid aims to identify the conserved motif of phytase from *A. niger*. The identity has a good result as shown before, with conserved histidine as a part of a catalytic site [4]. High identity showed a highly similar product as the template based on the presence of a conserved motif, indicating that the sequence is a part of histidine acid phosphates (HAP) [8]. Alanine replaces the glycine motif on phytase 49.GAQ46510.1 and did not present a conserved signature motif in the amino acid sequences indicating that the phytase may not be from these isolates of histidine phosphatase. This makes the active site molecule slightly stiffer but, more stable. In addition, alanine is more hydrophobic (as indicated by the greater hydropathy value) and larger relative molecule mass than glycine [28].

The present study found phytases have similarities in the mutations. These similarities are then taken into consideration in determining the candidate source of *A. niger* phytase. *A. niger* is a HAP enzyme with characteristic motif –RHGXRXP– and –HD– [11, 25]. Therefore, deliberate mutation can only be performed on amino acid X (unknown/other) which based on the result is shown by alanine and tyrosine. Both amino acids can be converted into uncharged or aromatic non-polar amino acids such as valine and phenylalanine [4, 28].

Phylogenetics based on *A. niger* phytase gene sequences showed these results were not much different from those shown by phylogenetic trees made based on amino acids. This is the sequence representative within a clade constitute reference [29]. Therefore, the candidate phytase gene source of *A. niger* 110.M94550.1 was obtained, which is the most identical gene source to *A. niger* 3k4p, as well as 48.2.BCMY01000003.1 and 92.JQ654450.1 that are the best representative of the gene source group that has certain characteristic mutations.

The three primer designs are quite identical with a low secondary structure. This also lowers mispriming due to complementation between primers or between forward and reverse primers such as self-annealing, hairpin, and nonspecific amplification [30]. The limitation of the primer design is the annealing temperature on primers was the same as the extension temperature. Annealing temperature ranges from 45 to 60 °C and 72 °C for extension temperature [31]. The high annealing temperature certainly causes the attachment to be not optimal because the primer is difficult to anneal [32]. Based on PCR simulation using Snapgene, all the designs still have mispriming possibilities. This can be caused by the presence of repetition of the same base in the sequence. Base repetition can also cause breathing of the primer [32]. These factors can reduce primer specificity and allow mispriming. However, the maximum amplicon result on FastPCR tells us that all the designs were able to fully bind to the template and produce products. This indicates that these designs yielded good results. The use of longer primers is one of the things that can be done to reduce the possibility of mispriming [30, 32]. We suggest these primers only be used to identify the source of the phytase gene. The primer usability to produce phytase should be directly tested first. Despite the limitation of the result, this study is an initial and important step to produce phytase using genetic and protein engineering in its implementation.

To summarize, as a result of the bioinformatics study it was found that the *A. niger* phytase has a stable protein structure. There are 14 *A. niger* phytases that have identities above 95%. *A. niger* 110. M94550.1 is the closest strain to the PDB template. Most of the *A. niger* phytase sequences are well conserved. The phytase gene source candidates obtained were *A. niger* 110.M94550.1, which was the only phytase that did not have mutations against the PDB template, and *A. niger* 48.2.BCMY01000003.1 and 92.JQ654450.1 was the best representative of the gene source group that had certain characteristics. Primer design in this study produced the best primer set with a forward primer length of 19 bp and a reverse primer with a length of 24 bp with 1404 bp amplicons. The produced primer designs were able to fully bind to the template and, however, still show some possibility of mispriming so it can only be used to identify the source of the phytase gene.

## Conclusions

Phytase *A. niger* has a good characteristic with stable protein and high identity. However, the resulting primer design still shows the possibility of mispriming. This indicates the high potential for further development, especially the production of phytase enzymes from *A. niger* as a feed additive using genetic engineering in an effort to develop the quality of animal feed in Indonesia. Therefore, this study needs to be studied further so that the potential can be explored and utilized optimally.

## Data Availability

The phytase sequence of the *A. niger* template is available in the Protein Data Bank (ID: 3k4p and 3k4q). The complete DNA sequence and the corresponding annotation of A. niger is available in the NCBI Genbank (accession number BCMY01000003.1; BCMY01000021.1; AB022700.1; JQ654450.1; JQ654449 0.1; AY426977.1; AY745739.1; L02421.1; JQ241266.1; EF197825.1; L20567.1; AY513749.1; M94550.1; JN196454.1; AF218813.1; L02420.1; EU786167.1) The complete amino acid sequence and the corresponding annotation of A. niger is available in the NCBI Genbank (accession number GAQ37582.1; GAQ37712.1; GAQ46510.1; BAA74433.1; AFJ79736 0.1; AFJ79735.1; AAR08366.1; AAU93518.1; AAA16898.1; AFE56108.1; ABM92786.1; AAA02934.1; AAS00648.1; AAA32705.1; AET71192.1; AAF25481.1; AAA16897.1; ACE79229.1).

## Abbreviations

A. niger: Aspergillus niger
HAP: Histidine acid phosphatase
PDB: Protein data bank
NCBI: National Center of Biotechnology Information
DNA: Deoxyribonucleic acid
CDS: Coding data sequence
SPDBV: Swiss Protein Data Bank Viewer
Bp: Base pair
aa: Amino acid
PCR: Polymerase chain reaction
IHS: Myo-inositol hexasulfate
IHP: Myo-inositol hexaphosphate
CPK: Creatine phosphokinase
Olc: Oligocalculator
P3 +: Primer 3 +
Tm: Melting temperature
TA: Annealing temperature
SA: Self-annealing
SC: Self complementary
C: Complementary
